# Shift of Nitrogen and Carbon Stable Isotopes in Temporary Pond Tadpoles Following the Decline of Large Mammalian Herbivores

**DOI:** 10.1002/ece3.73508

**Published:** 2026-04-14

**Authors:** Nick Ewald, Guillaume Demare, Julian Glos, Ulrich Struck, Mark‐Oliver Rödel

**Affiliations:** ^1^ Museum für Naturkunde – Leibniz Institute for Evolution and Biodiversity Science Berlin Germany; ^2^ Institute of Biology Freie Universität Berlin Berlin Germany; ^3^ Institute of Cell and Systems Biology Universität Hamburg Hamburg Germany

**Keywords:** anura, cascading effects, communities, savanna, temporary ponds

## Abstract

Nutrient cycling across ecosystems is driven by complex ecological interactions, potentially impacting environmental stability. Savanna ecosystem processes are highly driven by large mammalian herbivores, so removing them likely causes significant changes in nutrient cycling in both terrestrial and aquatic habitats. Aquatic organisms in temporary savanna ponds, such as amphibian larvae (i.e., tadpoles), are good models to study this potential indirect impact. In the savanna of Comoé National Park, Ivory Coast, large mammalian herbivores declined through two civil war periods in 2002 and 2011. This led to a subsequent increase in terrestrial and aquatic vegetation density, and an altered tadpole community composition. In this study, we used stable isotope analysis to examine potential shifts in energy sources and food web positioning of savanna tadpoles. We compared tadpole samples collected before (1995 and 1996) and after the wars (2014, 2018, and 2019) from two ponds. We detected a general decrease in δ^15^N among tadpoles, which we interpret as a consequence of a baseline shift in δ^15^N values following mammalian declines. There was no overall shift in δ^13^C signals, suggesting that changes in primary energy resources were minimal. Observed interannual changes in isotope signals were taxon‐specific, reflecting differences in tadpoles' morphologies and respective diets, as well as site‐specific, reflecting different pond characteristics. Our findings indicate the potential importance of large mammalian herbivores in maintaining savanna ecosystem stability.

## Graphical abstract

Our study investigates how the decline of large mammalian herbivores in Comoé National Park, Ivory Coast, influenced nutrient dynamics in temporary ponds. Using stable isotope analyses of tadpoles collected before (1995–1996) and after (2014–2019) the decline, we reveal a consistent decrease in δ^15^N values across multiple taxa, suggesting a system‐wide shift in nitrogen baseline likely resulting from reduced cross‐ecosystem nutrient inputs. Our results highlight the strong coupling between terrestrial and freshwater ecosystems and demonstrate the cascading consequences of large‐mammal declines for aquatic nutrient cycling.

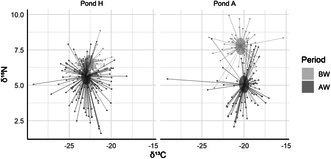

## 1 Introduction

The transfer of nutrients across ecosystem boundaries is both a key driver and a consequence of ecological processes. To understand how ecological stability is maintained, it is, therefore, essential to examine nutrient cycling, energy flows, and trophic interactions beyond single ecosystem types (Polis et al. 1997; Loreau et al. 2003). Many cross‐ecosystem flows take place at the terrestrial‐freshwater interface, driven by passive physical processes such as gravity and wind (Gounand et al. 2018). However, many terrestrial‐aquatic linkages are actively driven by animal movements, either at large spatial scales via seasonal migrations (Bauer and Hoye 2014), or because of smaller‐scale movements between adjacent ecosystems. In African savannas, for instance, hippopotamuses ( *Hippopotamus amphibius* ) transport terrestrially derived nutrients into adjacent rivers by grazing on land at night and defecating in the water during the day, thereby subsidizing aquatic ecosystems with significant amounts of carbon, nitrogen, and phosphorus (Masese et al. 2015; Subalusky et al. 2015). The loss or decline of species that mediate cross‐ecosystem nutrient flows can threaten the integrity of recipient ecosystems, sometimes to the point of complete ecosystem shifts (Croll et al. 2005).

The removal of large mammalian herbivores can lead to a wide range of ecological effects, including changes in nutrient cycling. In savanna ecosystems, large herbivores directly influence vegetation cover, structure, and diversity through grazing and browsing (Owen‐Smith 1988; Sankaran et al. 2005; Asner et al. 2009), and they help create and maintain nutrient‐rich patches in the landscape (Augustine et al. 2003; Winnie et al. 2008). They also control fuel load availability, and thus fire regime, which further determines vegetation dynamics and the spatial distribution of nutrients (Higgins et al. 2007; Holdo et al. 2009; Massad et al. 2024). In Mozambique's Gorongosa National Park, for example, the sharp human‐driven decline in large herbivore populations resulted in major changes in vegetation structure and the loss of ecosystem functions (Daskin et al. 2016; Correia et al. 2017). In addition, removing large terrestrial herbivores is expected to alter nutrient cycling in adjacent freshwater systems, both indirectly through changes in vegetation structure and composition (MacSween et al. 2019) and directly via reduced nutrient inputs (Subalusky et al. 2015). These cross‐ecosystem effects, however, remain little studied (Bakker et al. 2016).

In this study, we investigated how the decline of large mammalian herbivores may influence pond nutrient dynamics by looking at changes in stable isotope signatures of aquatic anuran larvae (i.e., tadpoles). We focused our investigation on temporary ponds, which, despite their small size, support important biodiversity (Céréghino et al. 2008; Snoeks et al. 2021). Temporary ponds are essential breeding habitats for a wide range of species with a biphasic life‐cycle. These species assimilate nutrients as aquatic larvae and transport them back to adjacent terrestrial habitats as metamorphs (Regester et al. 2008; Capps et al. 2015; Reinhardt et al. 2017; Fritz and Whiles 2018). Among these, anurans are an important group of pond‐dwelling organisms, because tadpoles can reach remarkably high densities, acting as both predominant primary consumers and abundant prey (Wilbur 1997). Tadpoles display a wide variety of diets, including detritivory, herbivory, omnivory, and even carnivory, which reflect different strategies in nutrient assimilation (Altig et al. 2007). However, tadpoles may shift their diets depending on biotic and abiotic conditions, such as competition and resource availability (Arribas et al. 2015). Nutrient cycling in ponds is therefore the outcome of nutrient inputs, for example, from large mammals, as well as trophic dynamics within ponds, which are influenced by abiotic and biotic conditions.

We conducted our study in Comoé National Park, a protected savanna area in Ivory Coast, West Africa, which experienced sharp declines of large mammal populations because of intensified poaching during two consecutive civil wars in 2002–2007 and 2010–2011 (Fischer 2004; Scholte et al. 2025). These declines were accompanied by major changes in temporary ponds, including an average threefold increase in pond vegetation cover and shifts in tadpole communities (Demare et al. 2022). By comparing tadpoles collected before and after mammalian declines, we had a unique opportunity to examine how the loss of large mammals (and associated habitat and community shifts) may be reflected in the stable isotope ratios of nitrogen (δ^15^N) and carbon (δ^13^C). Stable isotope ratios can provide insights into food web structure, but their interpretation requires distinguishing between two non‐mutually exclusive processes: changes in consumer trophic position and shifts in isotopic baselines. Although δ^15^N is commonly used to infer trophic position and δ^13^C to trace basal energy sources, isotopic shifts can also arise from changes at the base of the food web, such as altered nutrient inputs, primary production, or biochemical cycling (Peterson and Fry 1987; Post 2002; Layman et al. 2007). Because these different mechanisms cannot be easily disentangled, our study does not aim to isolate a single driver. Instead, we use stable isotope analysis as an integrative approach to detect ecosystem‐wide shifts in isotopic signatures, which may reflect both direct and indirect consequences of large mammal declines on nutrient dynamics in temporary ponds.

In this study, we investigated whether the decline of large mammal populations in Comoé National Park has altered the isotopic signatures of pond‐breeding anurans. We tested for (1) directional shifts at the community level between the two study periods at two different ponds, (2) interspecific differences in isotopic shifts, and (3) compared temporal (interannual) and spatial (between‐pond) variation in isotopic signatures.

## 2 Methods

### 2.1 Study Area and Data Collection

We conducted our study in Comoé National Park (CNP), northeast Ivory Coast, West Africa. The area consists of a mosaic of savanna and forest patches, as well as gallery forests along the Comoé River, which flows from North to South. Temporary savanna ponds in CNP fill up during the rainy season, from March until November, when they are essential breeding sites for over 30 anuran species (Rödel 1998, 2000). We collected tadpoles in pre‐war (BW: 1995 and 1996) and post‐war (AW: 2014, 2018, and 2019) years from two temporary ponds located in the southern part of CNP: Pond A (8.7259 N, 3.8425 W) and Pond H (8.7552 N, 3.7771 W), locally known as Aussichtsberg and Hyperolius ponds, respectively. A list of all study ponds included in previous studies can be found in Demare et al. (2022). Our study focuses on Pond A and H because they were the only ponds for which BW isotope data were available.

**FIGURE 1:**
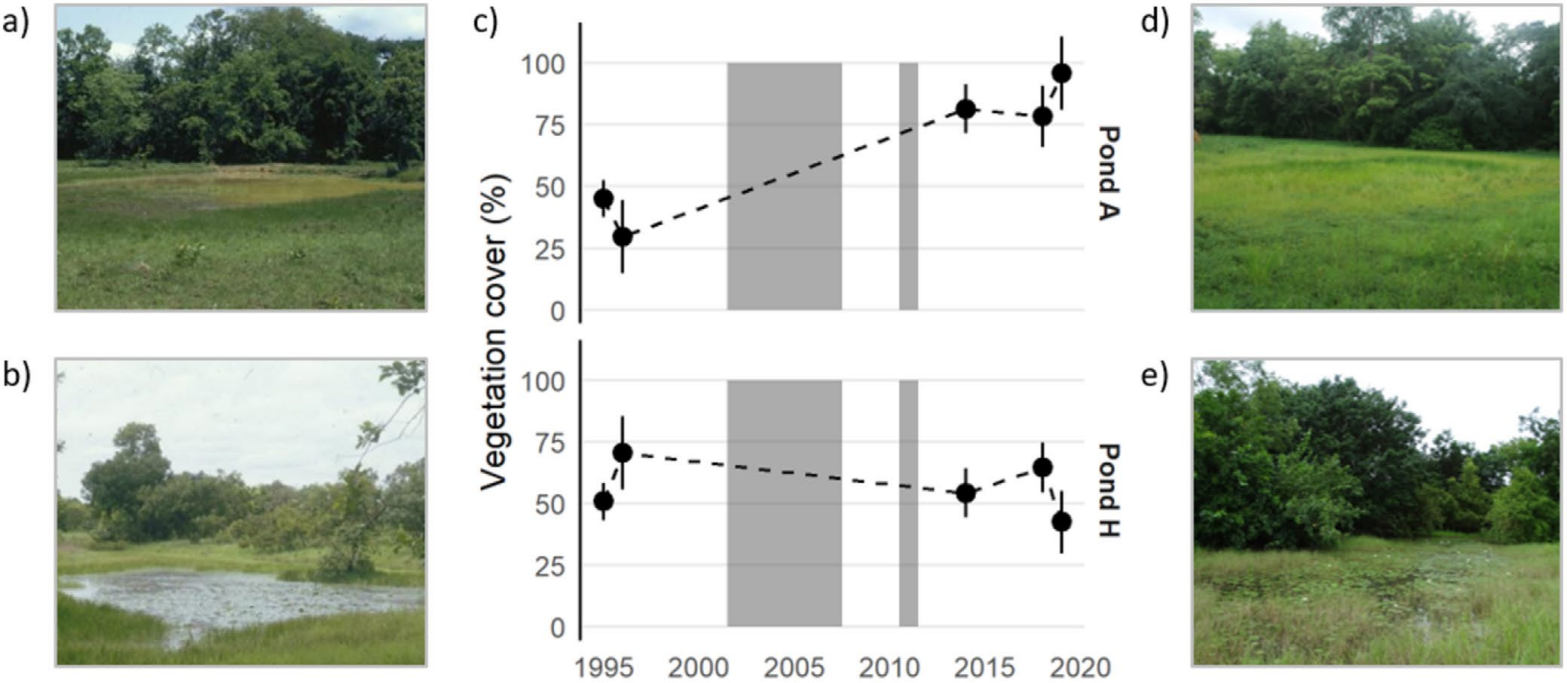
(a) Pond A in the pre‐war (BW) period; (b) Pond H in the BW period; (c) Average pond vegetation cover (Pond A and H) between sampling years; gray shaded areas indicate the two periods of civil unrest (2002–2007 and 2010–2011); (d) Pond A in the post‐war (AW) period; (e) Pond H in the AW period. Data in (c) was obtained from Demare et al. (2022).

Pond A is relatively shallow, with maximum water depth not exceeding 35 cm, and measures ca. 30 × 20 m. Pond H is deeper, with a maximum water depth of 55 cm, and measures ca. 55 × 15 m. In addition, Pond A is located near the top of a hill on a plateau, whereas Pond H is positioned in a depression isolated from other ponds, and becomes occasionally connected to the Comoé River during heavy rains. These connections in the rainy season via a temporary creek could potentially introduce external nutrients from a wider savanna area, but note that rainfall patterns in CNP do not appear to have changed significantly since the BW period (Demare et al. 2022). Figure 1 shows that Pond A experienced a significant increase in vegetation cover in the AW period, which is a trend experienced by most temporary ponds in the study area (Demare et al. 2022), whereas the vegetation in Pond H remained very similar.

Tadpoles were caught with dip nets, euthanized in a chlorobutanol solution, fixed in 4% formaldehyde upon collection, and subsequently transferred to 75% ethanol for long‐term preservation. They were identified on the basis of external morphology (see Rödel and Spieler 2000). Tadpoles of the following genera could not be reliably identified to species level and were therefore aggregated under the generic name (in parentheses, we give the number of species within that genus that are known from CNP; compare Rödel 1998): *Afrixalus* (3 spp.), *Hyperolius* (3 spp.), *Kassina* (4 spp.), *Phrynobatrachus* (5 spp.), and *Ptychadena* (7 spp.). Our analysis thus focused on nine taxa: four biological species ( *Hemisus marmoratus* , *Phrynomantis microps* , *Hoplobatrachus occipitalis* , *Hildebrandtia ornata* ) and the five aforementioned genera. One taxon, *Sclerophrys maculata*, was excluded because it is only sporadically found in temporary savanna ponds, and we did not collect samples for stable isotope analysis.

We obtained stable isotope data for 189 tadpole samples collected in the AW period, against 124 samples from the BW period. The final dataset comprised 126 samples for *Kassina* spp., 44 for *Hyperolius* spp., 39 for *Ptychadena* spp., 37 for *Afrixalus* spp., 22 for *Phrynomantis microps* , 20 for *Hemisus marmoratus* , 14 for *Phrynobatrachus* spp., 7 for *Hoplobatrachus occipitalis* , and 4 for *Hildebrandtia ornata* . Although *H. ornata* was collected in BW surveys, it was not detected in the AW period, and we only had a few samples from a single pond site in both sampling periods for *H. occipitalis* and *P. microps* . For these three taxa, the sample size was therefore too low to statistically estimate AW effects (see ‘Data analysis’ below). We provide a sampling summary by taxon, period, and site in Table 1.

**TABLE 1:** Summary of samples collected in the pre‐war (BW) and post‐war (AW) periods, in ponds A and H. Taxa for which sample size was large enough for at least one pond site (i.e., *N* ≥ 3) to quantify AW effects are indicated in bold.

	Pond H		Pond A	
	BW	AW	BW	AW
***Afrixalus* spp**.	8	16	4	9
***Hemisus marmoratus***	4	10	1	5
*Hildebrandtia ornata*	—	—	4	—
*Hoplobatrachus occipitalis*	2	—	4	1
***Hyperolius* spp**.	4	33	—	7
***Kassina* spp**.	26	51	24	25
***Phrynobatrachus* spp**.	4	6	4	—
***Phrynomantis microps***	15	—	2	5
***Ptychadena* spp**.	8	2	10	19

### 2.2 Stable Isotope Analysis

For stable isotope analyses, we extracted tail muscle tissue from the middle part of the tail axis of each tadpole. These samples were then dried in a drying oven at 40°C for at least 3 days. The dry samples (0.5–1 mg) were then placed in tin cups for analysis. Stable isotope analysis and concentration measurements of nitrogen and carbon were performed simultaneously with a THERMO/Finnigan MAT V isotope ratio mass spectrometer coupled to a THERMO Flash EA 1112 elemental analyzer via a THERMO/Fisher Conflo IV‐interface in the stable isotope laboratory of the Museum für Naturkunde, Berlin. Stable isotope ratios are expressed in the conventional delta notation (δ^13^C/δ^15^N) relative to atmospheric nitrogen (Mariotti 1983) and VPDB (Vienna PeeDee Belemnite standard). Standard deviation for repeated measurements of lab standard material (peptone) was generally better than 0.15 per mill (‰) for nitrogen and carbon, respectively. Standard deviations of concentration measurements of replicates of our lab standard were < 3% of the concentration analyzed. One δ^13^C measurement was largely deviating from all other data. This was most likely a measuring error, and the sample was therefore excluded from the analysis.

### 2.3 Data Analysis

First, we used a mixed effects modeling approach (GLMM) to estimate overall post‐war changes in δ^15^N and δ^13^C at each pond site, averaged across all anuran taxa. We included genus as a random effect in each model. Model fixed effects included the following categorical variables: sampling period (BW and AW), pond site (A and H), as well as an interaction term between the two. Initial model diagnostics indicated unequal variance in residuals across all levels of each categorical predictor, so we incorporated observation‐level weights inversely proportional to the estimated residual variance for each combination of predictors (period and site). Weights ($\omega_{i}$) were calculated as: $\omega_{i}=\frac{1}{{\overset{`}{\sigma }}_{g}^{2}}$, where ${\overset{`}{\sigma }}_{g}^{2}$ is the estimated residual variance for group g, defined by unique combinations of period and site. Residual variance estimates were obtained from an unweighted model and assigned to each observation on the basis of its respective group.

Second, we investigated taxon‐specific differences in isotopic response by fitting a separate linear model (LM) for each taxon that comprised at least three samples for at least one pond site in both sampling periods. We therefore excluded the following taxa: *H. ornata* (only collected in the BW period), *H. occipitalis* (only a single AW sample), and *P. microps* (no samples from Pond H in the AW period and only two samples from Pond A in the BW period). For *Afrixalus*, *Kassina*, and *Ptychadena*, we were able to include both sampling periods (BW and AW) and ponds (H and A) as fixed effects, and we tested the significance of the interaction between the two. For *H. marmoratus* , *Hyperolius*, and *Phrynobatrachus*, we used data exclusively from Pond H because not enough tadpoles were collected from Pond A in the BW period. We, therefore, did not include pond site as a model predictor. However, all models included body size as an additional fixed effect to account for the potential influence of ontogenetic shifts in δ^15^N and δ^13^C, as this was previously reported for tadpoles from the same study area (Glos et al. 2017).

Third, we explored interannual fluctuations in isotopic levels, rather than aggregating data solely by sampling period (BW vs. AW) as in the previous steps. By examining variation between years, we aimed to distinguish consistent directional changes potentially attributable to mammal declines from natural temporal variability, and to assess whether taxa and sites responded synchronously or exhibited divergent interannual trajectories.

We tested the fixed and random effects structures of each model using likelihood ratio tests and Akaike's Information Criterion (AIC) after refitting all models with Maximum Likelihood estimation. In addition, we assessed the significance of each model fixed effect using Wald tests at the 0.05 level of significance. Model residuals were examined to confirm that weighting reduced heteroscedasticity. All statistical analyses were conducted in R version 4.4.2. We fitted mixed effects models using the package *lme4* (Bates et al. 2015) and visualized model results using *ggplot2* (Wickham 2016).

## 3 Results

### 3.1 Post‐War Isotopic Shifts

**FIGURE 2:**
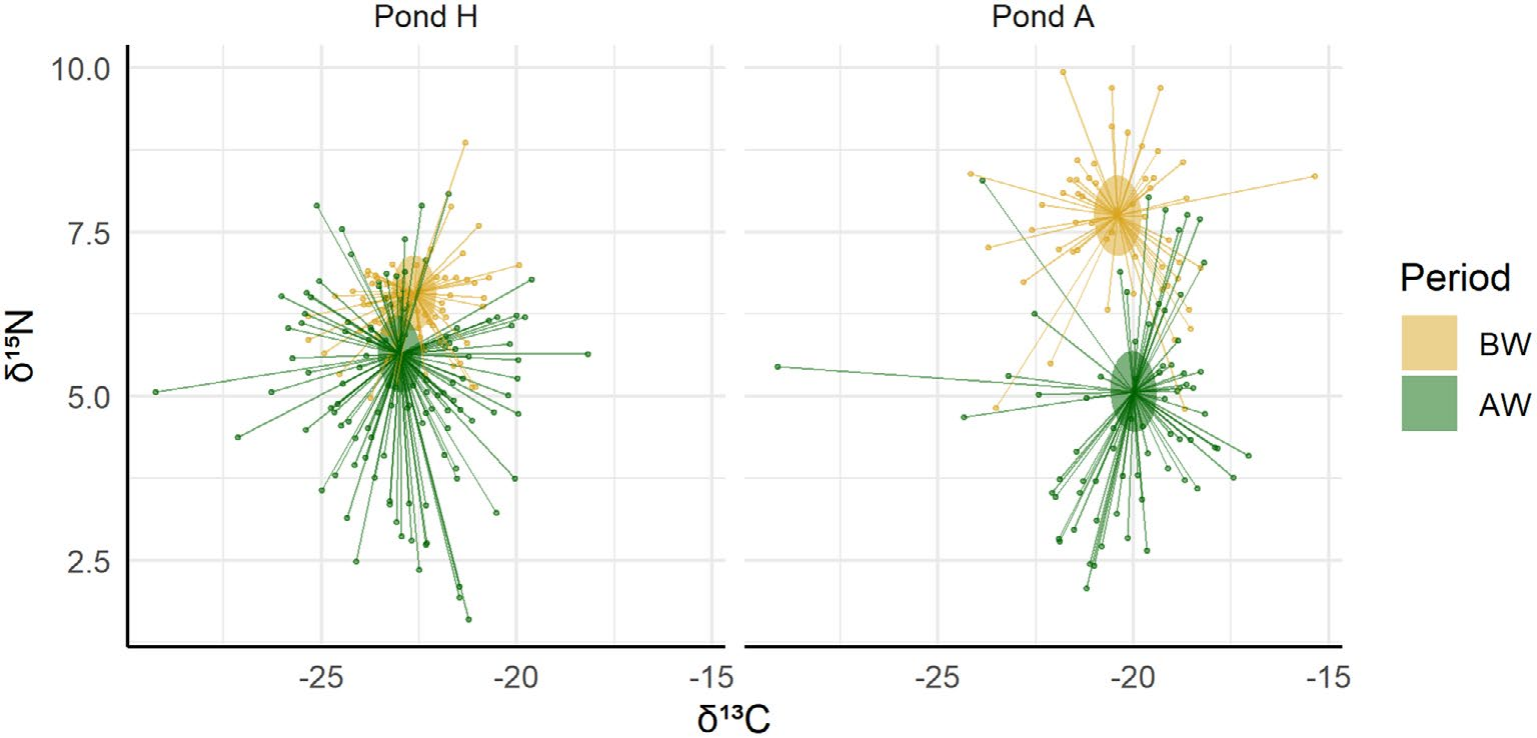
Pre‐war (BW in yellow) and post‐war (AW in green) estimates of δ^15^N and δ^13^C levels (*y*‐ and *x*‐axis, respectively), averaged across all tadpole taxa, with 95% confidence intervals as ellipses. Each point corresponds to observed values for a given sample.

Figure 2 and Table 2 show an overall significant post‐war (AW) decrease in δ^15^N (t_303_ = −6.76, *p* < 0.001), but no significant change in δ^13^C (t_307_ = −1.46, *p* = 0.144), averaged across all measured tadpole genera for Pond H. The AW decrease in δ^15^N was more pronounced for Pond A, with on average 1.75‰ less than Pond H in the AW period (t_304_ = −7.10, *p* < 0.001). However, pre‐war (BW) δ^15^N levels were significantly higher in Pond A (t_307_ = 7.31, *p* < 0.001). The same was true for BW δ^13^C levels (t_309_ = 7.63, p < 0.001). We also noted that Pond A showed slightly higher δ^13^C values than Pond H in the AW period (t_309_ = 2.03, *p* = 0.044), but the significance of this interaction was only marginal and should therefore be treated with caution, especially given that we did not find strong support for a significant AW effect concerning δ^13^C.

**TABLE 2:** Summary of δ^15^N and δ^13^C GLMM results. For each model, we included genus as a random effect (1|genus), the significance of which was tested with a log‐likelihood ratio (LLR) test. We provide the corresponding marginal and conditional *R* ^2^ (*R* ^2^m and *R* ^2^c, respectively), and the difference in AIC compared to a null model with intercept only. For each model fixed effect, we provide the estimate, standard error (SE), *t*‐value, degrees of freedom (df), and *p*‐value. Significant fixed and random effects are highlighted in bold (*α* = 0.05).

		Estimate	SE	*t*‐value	LLR	df	*p*	*R* ^2^m	*R* ^2^c	AIC	ΔAIC
**δ** ^**15**^ **N**					162.31	3	0	0.33	0.60	916.2	−149.7
	**1|genus**				69.67	1	< 0.001				−61.1
	Intercept^a^	6.57	0.29	22.49		7	< 0.001				
	**periodAW**	−0.93	0.14	−6.76		303	< 0.001				
	**pondA**	1.18	0.16	7.31		308	< 0.001				
	**periodAW:pondA**	−1.75	0.25	−7.10		304	< 0.001				
**δ** ^**13**^ **C**					157.89	3	0	0.39	0.47	1192.0	−148.7
	**1|genus**				18.00	1	< 0.001				−11.9
	Intercept^a^	−22.63	0.29	−77.14		16	< 0.001				
	periodAW	−0.37	0.25	−1.46		307	0.144				
	**pondA**	2.23	0.29	7.63		309	< 0.001				
	**periodAW:pondA**	0.77	0.38	2.03		309	0.044				

^a^ Intercept taken for pond H and sampling period BW.

Log‐likelihood ratio tests showed that including taxon identity (i.e., genus) as a random effect significantly improved the fit of both δ^15^N and δ^13^C models (see Table 2). This was especially true for δ^15^N (χ^2^_1_ = 69.67, *p* < 0.001), with conditional R^2^ equal to 0.60, compared to a marginal R^2^ equal to 0.33. Different tadpole taxa also differed in δ^13^C, but not as strongly (χ^2^_1_ = 18.00, *p* < 0.001), with only a small difference between conditional and marginal R^2^. The associated change in AIC was −61.1 for δ^15^N and −11.9 for δ^13^C.

In summary, the model results brought evidence for an overall decrease in δ^15^N, averaged across all taxa, as well as genus‐level and site‐specific differences in isotopic values for both δ^15^N and δ^13^C. We did not find strong evidence for significant AW changes in δ^13^C. Below we explore further taxon‐specific AW effects.

### 3.2 Interspecific and Interannual δ^15^N Responses

**FIGURE 3:**
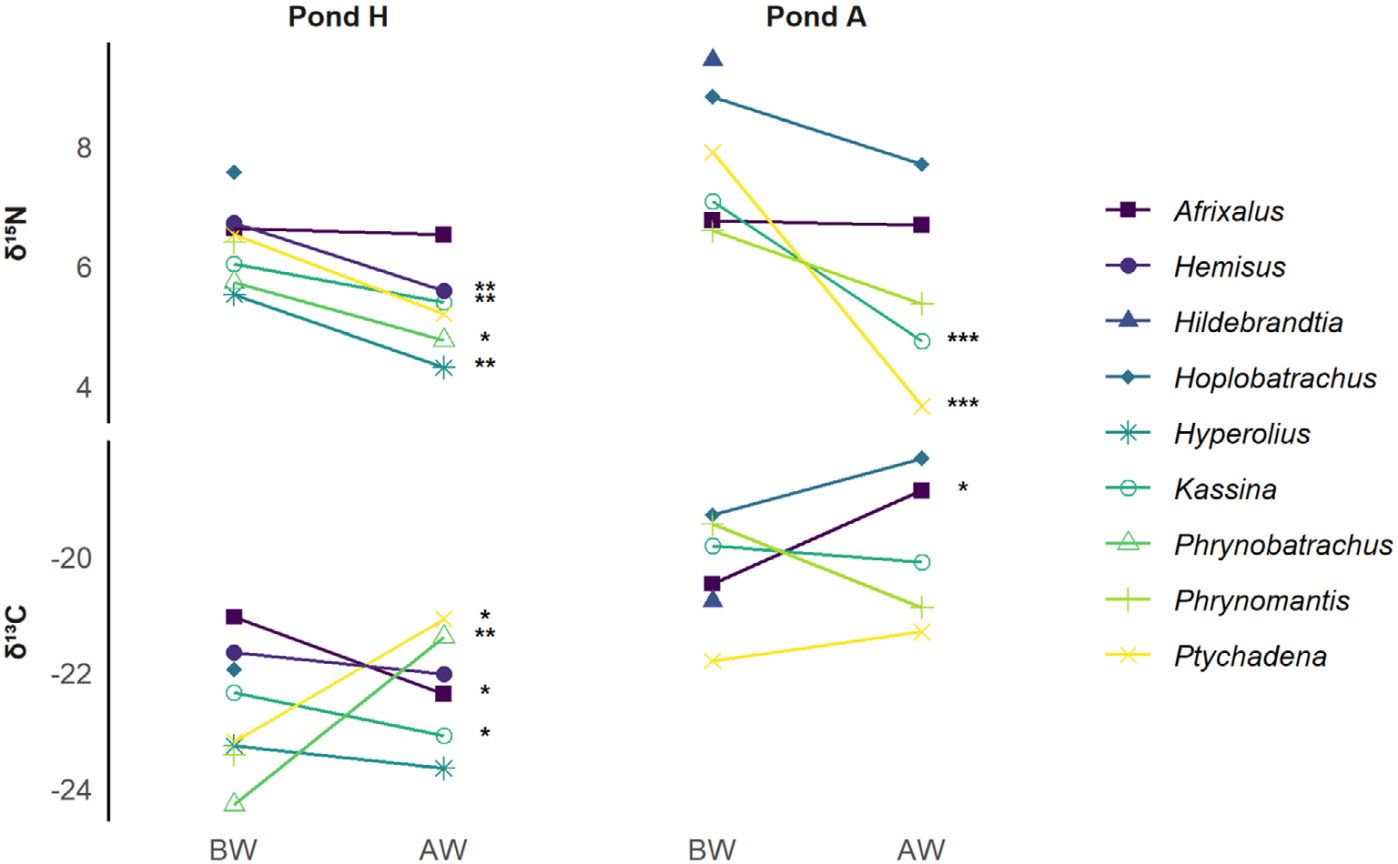
Pre‐war (BW) and post‐war (AW) shifts in δ^15^N and δ^13^C, for each tadpole taxon and pond site (A and H), estimated by fitting a GLM with median observed body size. Statistically significant AW shifts are denoted with the following symbols: * = *p* < 0.05; ** = *p* < 0.01; *** = *p* < 0.001. For taxa with low sample size, that is, *Hildebrandtia ornata* , *Hoplobatrachus occipitalis* , and *Phrynomantis microps* , BW and AW estimates were obtained from mean values.

**FIGURE 4:**
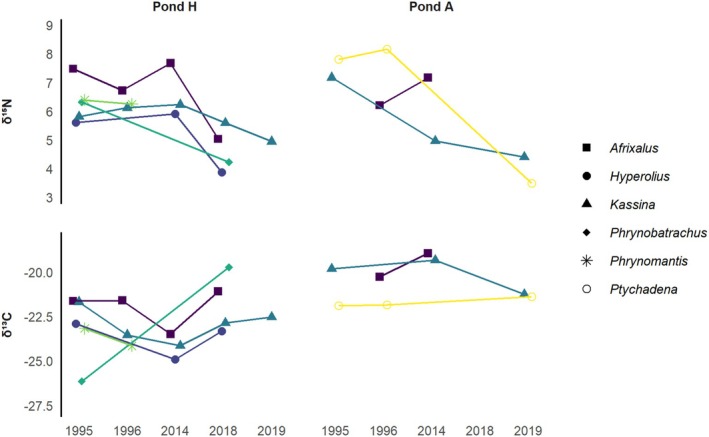
Estimated isotopic values (δ^15^N and δ^13^C) for each tadpole taxon, sampling year, and pond site (A and H). We only included taxa that were sampled in at least two different years and years for which there were at least three data points.

Despite finding an overall AW decrease in δ^15^N, there was variation in isotopic responses between taxa and sites (Figure 3), as well as interannual variation (Figure 4). Corresponding model results and observed δ^15^N values are detailed in Supplementary Information S1 (Tables S1, S3, S4, S6, and Figures S1 and S2, respectively). The greatest AW decrease in δ^15^N was experienced by *Ptychadena* in Pond A (−4.24‰, t_34_ = −15.15, *p* < 0.001), which also had among the highest BW levels of δ^15^N. This significant AW decrease appears to have taken place before 2014 (Figure S2), but we did not have enough 2014 observations to statistically test this. *Kassina* showed a similar significant reduction in AW levels of δ^15^N in Pond A (−2.34‰, t_121_ = −7.04, *p* < 0.001), with values significantly lower in 2014 compared to 1995 (−2.21‰, t_45_ = −7.16, p < 0.001), and statistically similar between AW years (2014 and 2019).

*Kassina* also showed a drop in δ^15^N values in Pond H in 2018, although not statistically different from 2014 (−0.64‰, t_71_ = −2.44, *p* = 0.116), and significantly so in 2019 compared to both 2014 (−1.28‰, t_71_ = −4.25, *p* < 0.001) and 2018 (−0.65‰, t_71_ = −2.88, *p* = 0.041). With the exception of *Afrixalus*, all other taxa showed a slight but significant AW decrease in δ^15^N in Pond H: *Hemisus marmoratus* (−1.13‰, t_11_ = −4.17, *p* = 0.002), *Hyperolius* (−1.22‰, t_34_ = −3.57, *p* < 0.001), and *Phrynobatrachus* (−0.97‰, t_7_ = −2.48, *p* = 0.042). As in *Afrixalus*, however, δ^15^N values for 2014 were not statistically different from BW years (i.e., 1995 and/or 1996). *Hyperolius* showed a significant decrease in δ^15^N from 2014 to 2018 (−2.03‰, t_32_ = −4.36, *p* < 0.001), and *Phrynobatrachus* only showed significantly lower δ^15^N values in Pond H in 2018 compared to 1995 (−2.08‰, t_5_ = −3.27, *p* < 0.022). In the case of *Afrixalus* δ^15^N values in Pond H dropped significantly in 2018 (−2.65‰, t_17_ = −4.51, *p* = 0.002), but we did not detect a statistically significant shift in 2014 compared to BW years in either Pond H or A.

### 3.3 Interspecific and Interannual δ^13^C Responses

In contrast to the consistent AW decreases observed in δ^15^N, Figure 3 highlights that AW shifts in δ^13^C were more variable and showed no clear directional pattern across taxa and pond sites. Corresponding model results and observed δ^13^C values are detailed in Data S1 (Tables S2, S3, S5, S7, and Figures S1 and S3, respectively) *Phrynobatrachus* was one of few taxa to show a significant AW increase in δ^13^C values (+2.90‰, t_7_ = 3.86, *p* = 0.006), as measured in Pond H. Similarly, *Ptychadena* showed a significant AW increase in the same pond (+2.12‰, t_34_ = 2.56, *p* = 0.015), and *Afrixalus* in Pond A (+1.61‰, t_32_ = 2.35, *p* = 0.025). Interestingly, δ^13^C values for *Afrixalus* significantly decreased at the other pond site, that is, Pond H (−1.33‰, t_32_ = −2.57, *p* = 0.015). This trend corresponds to a significant decrease in 2014 compared to 1996 (−1.89‰, t_17_ = −3.29, *p* = 0.020), despite a significant increase from 2014 to 2018 (+2.41‰, t_17_ = 4.26, *p* = 0.003). We measured a similar AW decrease for *Kassina* in Pond H (−0.75‰, t_121_ = −1.99, *p* = 0.050), including significantly lower δ^13^C values between 1995 and 1996 (−1.86‰, t_71_ = −3.61, *p* = 0.005), no significant changes between 1996 and 2014, and values in 2019 that were statistically higher compared to 2014 (+1.61‰, t_71_ = 4.20, *p* < 0.001). We observed similar trends in *Hyperolius*, but differences were not statistically significant.

## 4 Discussion

In this study, we investigated changes in isotopic signatures of nitrogen (δ^15^N) and carbon (δ^13^C) of aquatic tadpoles, following significant declines of large mammalian herbivores in Comoé National Park, Ivory Coast. Our results show that the loss of large mammalian herbivores has coincided with reduced δ^15^N signatures in temporary savanna ponds among tadpoles. This is consistent with previous studies showing that large mammalian herbivores have an ecosystem‐wide influence on biogeochemical cycles, often transferring nutrients to aquatic systems (McNaughton et al. 1997; Semmartin and Oesterheld 2001; Bump et al. 2009; Masese et al. 2015; Subalusky et al. 2015; Bakker et al. 2016). The extent to which δ^15^N values decreased, however, was taxon‐ and site‐specific.

### 4.1 Community‐Wide Decrease in δ^15^N

The consistent and significant decrease in δ^15^N values in post‐war (AW) samples strongly suggests a system‐wide shift since the pre‐war period (BW). With the exception of *Afrixalus* (discussed below), all taxa exhibited this trend. The direction and magnitude of change were remarkably similar across taxa, especially in Pond H, on the basis of the BW–AW estimates that we obtained for five out of nine taxa. The decrease in δ^15^N in Pond H was not statistically significant for *Ptychadena*, but this was most likely because of low statistical power. Overall, it is unlikely that all taxa simultaneously reduced their trophic position since the BW period, which would otherwise explain lower δ^15^N values (Post 2002; Anderson and Cabana 2007). We note that tissue preservation in formaldehyde and ethanol may also alter isotopic profiles (Barrow et al. 2008), but all samples were treated equally and the effects that we measured go beyond what would be expected from preservation effects alone. We, therefore, interpret the overall decrease in δ^15^N values as evidence of a baseline δ^15^N shift.

Baseline changes in δ^15^N are commonly linked to variations in nitrogen inputs, such as eutrophication (Savage et al. 2004), or differences in biological nitrogen fixation (Dorado et al. 2012). Nitrogen inputs from animal waste are typically enriched in δ^15^N (10‰–20‰), whereas nitrogen derived from other sources, such as atmospheric nitrogen, typically has lower δ^15^N values (2‰–8‰) (Heaton 1986). As such, δ^15^N values of aquatic primary consumers are positively correlated with total nitrogen loading and source (Vander Zanden et al. 2005). In our study system, large mammalian herbivores are known to frequently use temporary ponds for drinking and mud‐bathing, so their BW‐AW decline likely reduced nitrogen input to ponds via defecation, which we propose as the most plausible explanation for the observed community‐wide decrease in δ^15^N values. Although dietary shifts in some taxa cannot be ruled out, assessing AW trophic changes would require precise estimates of baselines in δ^15^N values (Post 2002). This was not possible in our case because of a lack of isotopic data from pond vegetation during the BW period.

### 4.2 Taxon‐ and Site‐Specific δ^15^N Responses

Compared to other taxa, *Afrixalus* spp. appeared to maintain a high δ^15^N signature in the AW period at both pond sites. This may reflect the diet of *Afrixalus* tadpoles, given they are at least partially carnivorous and are known to prey on anuran eggs, other tadpoles (Rödel 1998; Griesbaum et al. 2025), and mosquito larvae (Minter et al. 2004). Their partial carnivorous diet aligns with our finding that *Afrixalus* tadpoles exhibited, on average, higher δ^15^N values than most other taxa. In the case of *Afrixalus*, allochthonous sources of nitrogen, for example, from mosquito larvae, may, therefore, act as a trophic buffer, dampening the influence of baseline shifts. The same may be true for *Hoplobatrachus occipitalis* and *Hildebrandtia ornata* , which have carnivorous tadpoles and are characterized by high δ^15^N signatures (Rödel 1998; compare Figure 3), but we did not have enough BW‐AW data for those two species to confirm this.

The apparent lack of δ^15^N decline in *Afrixalus* during the AW period could also be driven by the relatively higher δ^15^N values measured in 2014 (an AW year), despite this slight increase not being statistically significant compared to BW years. However, *Afrixalus* did show a significant decrease in 2018 compared to 2014. In fact, other taxa in Pond H, including *Kassina* spp. and *Hyperolius* spp., also showed δ^15^N levels in 2014 similar to those recorded during the BW period, followed by a significant decrease in subsequent AW years. Together, these results may suggest that a baseline decrease in δ^15^N at Pond H only occurred after 2014. To better understand differences in δ^15^N baseline levels between sampling years would require data on factors that are known to influence nitrogen inputs to ponds, such as exact site‐specific occurrence of large mammalian herbivores. That said, the apparent lack of an AW increase in vegetation cover in Pond H could indicate that mammalian occurrence at that particular pond may not have changed as dramatically since the BW period, compared to Pond A, which may in turn be reflected in the extent to which δ^15^N values decreased. Interannual fluctuations in δ^15^N levels detected in tadpoles may be driven by the timing of visits by large mammals, which can differ between sites as a function of mammal population size or habitat use (e.g., geographic occupancy changes due to poaching pressure). Because nitrogen inputs from mammals can be incorporated into aquatic food webs within weeks (Ashkenas et al. 2004), isotope signatures in tadpoles will reflect whether mammals were present shortly before or during a given sampling period. As a result, year‐to‐year variation may sometimes reflect chance, depending on whether sampling coincided with mammal use of a pond. In contrast, at sites where mammal presence (or absence) is more consistent across years, δ^15^N values should better represent longer‐term baselines rather than stochastic variation.

Interestingly, *Ptychadena* spp. in Pond A exhibited a relatively high δ^15^N signature in the BW period, even compared to *Afrixalus* (e.g., in 1996), and experienced a sharp decline in the AW period. On average, δ^15^N values of Pond A were consistently higher than those of Pond H in the BW period, as also reflected in the relatively high δ^15^N of *Kassina* tadpoles in 1995. This might indicate that elevated herbivore activity in the BW period contributed to a higher baseline in δ^15^N at this site. However, some of the observed differences in isotopic signatures may also reflect shifts in species composition within genera. Notably, *Ptychadena* tadpoles could not be reliably identified to species level, which constitutes a limitation of our study. If different *Ptychadena* species with distinct diets or habitat‐use patterns occurred in BW versus AW samples, this may have contributed to variation in δ^15^N values. Nonetheless, we expect this effect to be limited, as congeners in our study are morphologically similar, especially in their oral disc morphology (McDiarmid and Altig 1999; Rödel 2000), and thus likely have similar diets and isotopic values for δ^13^C and δ^15^N (Vences et al. 2016).

### 4.3 Potential Ecological Implications of Lower δ^15^N

Tadpoles play an important role in bioturbation, energy flow, and nutrient cycling in many aquatic food webs as primary and secondary consumers (Montaña et al. 2019). We found that δ^15^N in tadpoles decreased by approximately 1‰–3‰ for most taxa between the BW and AW periods. Using a standard enrichment factor of 3.4‰ per trophic level (Post 2002), this would correspond to a decline of 0.3–0.9 trophic levels. Because such a simultaneous shift across multiple taxa is unlikely, this supports our interpretation that the observed changes primarily reflect a shift in baseline δ^15^N rather than widespread dietary shifts. Although tadpoles probably did not all shift their trophic position, changes of this magnitude may nonetheless be biologically meaningful, especially if they are a consequence of nitrogen limitation. Although we do not have the necessary data to explore this aspect, nitrogen limitation has been shown to slow growth rates, reduce body size, and delay metamorphosis (Stoler and Relyea 2013; Stephens et al. 2017). This could be critical in savanna ecosystems where temporary ponds may desiccate before tadpoles complete their development (Smith 1983; Travis et al. 1985; Newman 1988). To link a drop in δ^15^N values to fitness would require directly measuring nutrient loading, while also measuring life‐history outcomes. In addition, pond‐breeding anurans export nutrients and energy to terrestrial environments during metamorphosis, similar to patterns described for emerging aquatic insects (Scharnweber et al. 2014), so altering nutrient inputs to temporary ponds could also bear consequences across ecosystem boundaries.

### 4.4 Variable δ^13^C Responses

We found no overall significant change in δ^13^C across the study period. As such, pond vegetation may still be dominated by C3 plants, despite ponds in the study area having experienced a drastic AW increase in vegetation cover (Demare et al. 2022). Indeed, the isotopic signature of a C3‐dominated system generally ranges between −24‰ and −33‰ (O'Leary 1981), which matches the δ^13^C levels that we measured at both pond sites in both sampling periods. The change of δ^13^C was less directional among taxa than for δ^15^N, despite some significant shifts in the AW period. For example, δ^13^C values for *Afrixalus* significantly decreased in Pond H, but significantly increased in Pond A. The outcome on carbon stable isotopes may, therefore, be highly context‐dependent, with different taxa in different environments showing distinct responses.

This context‐dependency likely arises because tadpoles differ in their feeding strategies, microhabitat preferences, and can show substantial niche differentiation in terms of carbon sources (Schiesari et al. 2009). The pond habitat changes that took place may have resulted in some species altering their use of the pond habitat, potentially shifting carbon sources in response. However, changes in nutrient inputs, potentially mediated by large mammals, can also have an effect on levels of δ^13^C (Hill et al. 2008). Although we cannot directly confirm this mechanism, it is worth noting that interannual changes in δ^15^N seemed to mirror those of δ^13^C (as an inverse relationship; compare Figure 4), especially in Pond H for most taxa. Carbon and nitrogen dynamics may be linked, even though we found δ^13^C changes to be less significant and less directional among taxa. To better understand these dynamics, future research could incorporate gut content analyses, either through morphological identification or DNA metabarcoding, to clarify diet composition and test for differences in carbon sources.

## 5 Conclusion

Our findings suggest that the decline of large mammalian herbivores in Comoé National Park is associated with a consistent decrease in δ^15^N values across multiple tadpole taxa in temporary savanna ponds, probably as a result of reduced nitrogen inputs to water bodies and lower baseline δ^15^N. Although δ^13^C values showed taxon‐ and site‐specific responses, they highlight the complex and context‐dependent nature of carbon dynamics in these systems. Together, these findings underscore the potential role of large‐bodied terrestrial mammals in shaping nutrient cycling in aquatic habitats and emphasize the ecological coupling between terrestrial and freshwater ecosystems. The observed isotopic shifts have potential implications for anuran development, population dynamics, and broader ecosystem functioning, particularly in nutrient‐limited water bodies. Understanding how amphibian communities may respond to altered nutrient regimes is crucial, not only for their conservation but also for better understanding cascading effects across ecosystem boundaries. Future work should aim to disentangle dietary shifts from baseline changes, incorporate direct measures of nutrient inputs and mammal activity, and further explore the functional roles of amphibians in biogeochemical cycles. Long‐term monitoring of ecosystems along disturbance and recovery gradients would also provide invaluable insights into these dynamic processes.

## Author Contributions

**Nick Ewald:** conceptualization (equal), data curation (equal), formal analysis (equal), investigation (equal), methodology (equal), project administration (equal), software (equal), validation (equal), visualization (equal), writing – original draft (equal), writing – review and editing (equal). **Guillaume Demare:** conceptualization (equal), data curation (equal), formal analysis (equal), investigation (equal), methodology (equal), project administration (equal), software (equal), validation (equal), visualization (equal), writing – original draft (equal), writing – review and editing (equal). **Julian Glos:** methodology (supporting), writing – review and editing (equal). **Ulrich Struck:** methodology (equal), resources (supporting), validation (supporting), writing – review and editing (equal). **Mark‐Oliver Rödel:** conceptualization (equal), funding acquisition (lead), methodology (equal), project administration (equal), resources (lead), supervision (lead), validation (supporting), writing – original draft (supporting), writing – review and editing (equal).

## Funding

This work was supported by Deutsche Forschungsgemeinschaft, Deutsche Gesellschaft für Herpetologie und Terrarienkunde, ‘Innovation‐Fond’ of the Museum für Naturkunde, Berlin, and Deutscher Akademischer Austauschdienst.

## Conflicts of Interest

The authors declare no conflicts of interest.

## Supporting information


**Data S1:** Model fitting and results.
**Figure S1:** Observed isotope values of δ^13^C (x‐axis) and δ^15^N (y‐axis) for each taxon and pond site. Pre‐war (BW) and post‐war (AW) samples are shown in yellow and green, respectively.
**Figure S2:** Observed isotope values of δ^15^N and corresponding violin plots for each taxon, sampling year, and pond site.
**Figure S3:** Observed isotope values of δ^13^C and corresponding violin plots for each taxon, sampling year, and pond site.
**Table S1:** Summary of linear models to estimate the effects of body size (cm), sampling period (BW vs. AW), pond site (A vs. H), and the interaction of sampling period and pond site, on the δ^15^N signature of each tadpole taxon. Significant predictors are highlighted in bold (α = 0.05). For each model parameter, we provide the estimate and standard error (SE). We tested the significance of each model predictor using a Wald test, and we performed a log‐likelihood ratio (LLR) test to compare each full model to the null model with intercept only, with corresponding degrees of freedom (DF), R^2^, and change in AIC.
**Table S2:** Summary of linear models to estimate the effects of body size (cm), sampling period (BW vs. AW), pond site (A vs. H), and the interaction of sampling period and pond site, on the δ^13^C signature of each tadpole taxon. Significant predictors are highlighted in bold (α = 0.05). For each model parameter, we provide the estimate and standard error (SE). We tested the significance of each model predictor using a Wald test, and we performed a log‐likelihood ratio (LLR) test to compare each full model to the null model with intercept only, with corresponding degrees of freedom (DF), R^2^, and change in AIC.
**Table S3:** Significance of each AW change in isotopic levels (δ^15^N and δ^13^C) for each pond (A vs. H) and tadpole taxon, compared to the BW baseline. Significant AW shifts are highlighted in bold (α = 0.05). For each test, we provide the standard error (SE), number of degrees of freedom (DF), T‐value, and corresponding P‐value. For taxa with low sample size, that is, *H. ornata* , *H. occipitalis* , and *P. microps* , BW and AW estimates correspond to the mean values.
**Table S4:** Summary of linear models to estimate the effects of body size (cm) and sampling year on the δ^15^N signature of each tadpole taxon at each pond site. Significant predictors are highlighted in bold (α = 0.05). For each model parameter, we provide the estimate and standard error (SE). We tested the significance of each model predictor using a Wald test, and we performed a log‐likelihood ratio (LLR) test to compare each full model to the null model with intercept only, with corresponding degrees of freedom (DF), R^2^, and change in AIC.
**Table S5:** Summary of linear models to estimate the effects of body size (cm) and sampling year on the δ^13^C signature of each tadpole taxon at each pond site. Significant predictors are highlighted in bold (α = 0.05). For each model parameter, we provide the estimate and standard error (SE). We tested the significance of each model predictor using a Wald test, and we performed a log‐likelihood ratio (LLR) test to compare each full model to the null model with intercept only, with corresponding degrees of freedom (DF), R^2^, and change in AIC.
**Table S6:** Post hoc test to compare pairwise year differences in δ^15^N for each tadpole taxon at each pond site (A and H). Significant pairwise differences are highlighted in bold (α = 0.05). For each test, we provide the standard error (SE), number of degrees of freedom (DF), T‐ratio, and corresponding *p*‐value.
**Table S7:** Post hoc test to compare pairwise year differences in δ^13^C for each tadpole taxon at each pond site (A and H). Significant pairwise differences are highlighted in bold (α = 0.05). For each test, we provide the standard error (SE), number of degrees of freedom (DF), T‐ratio, and corresponding *p*‐value.

## Data Availability

The data and code are provided as private‐for‐peer review using the following link: https://figshare.com/s/91027868e2419b3c691f. Upon acceptance of the manuscript, the data and code will be made publicly available on Figshare under the following DOI: https://doi.org/10.6084/m9.figshare.30705101.
