# Supplementary material for: Shift of Nitrogen and Carbon Stable Isotopes in Temporary Pond Tadpoles Following the Decline of Large Mammalian Herbivores
Source: Ecol Evol. 2026 Apr 14;16(4):e73508. doi: 10.1002/ece3.73508 (PMC13077444; doi:10.1002/ece3.73508)
Supplement: Supplementary file 1 — Data S1: Model fitting and results. Figure S1: Observed isotope values of δ13C (x‐axis) and δ15N (y‐axis) for each taxon and pond site. Pre‐war (BW) and post‐war (AW) samples are shown in yellow and green, respectively. Figure S2: Observed isotope values of δ15N and corresponding violin plots for each taxon, sampling year, and pond site. Figure S3: Observed isotope values of δ13C and corresponding violin plots for each taxon, sampling year, and pond site. Table S1: Summary of linear models to estimate the effects of body size (cm), sampling period (BW vs. AW), pond site (A vs. H), and the interaction of sampling period and pond site, on the δ15N signature of each tadpole taxon. Significant predictors are highlighted in bold (α = 0.05). For each model parameter, we provide the estimate and standard error (SE). We tested the significance of each model predictor using a Wald test, and we performed a log‐likelihood ratio (LLR) test to compare each full model to the null model with intercept only, with corresponding degrees of freedom (DF), R2, and change in AIC. Table S2: Summary of linear models to estimate the effects of body size (cm), sampling period (BW vs. AW), pond site (A vs. H), and the interaction of sampling period and pond site, on the δ13C signature of each tadpole taxon. Significant predictors are highlighted in bold (α = 0.05). For each model parameter, we provide the estimate and standard error (SE). We tested the significance of each model predictor using a Wald test, and we performed a log‐likelihood ratio (LLR) test to compare each full model to the null model with intercept only, with corresponding degrees of freedom (DF), R2, and change in AIC. Table S3: Significance of each AW change in isotopic levels (δ15N and δ13C) for each pond (A vs. H) and tadpole taxon, compared to the BW baseline. Significant AW shifts are highlighted in bold (α = 0.05). For each test, we provide the standard error (SE), number of degrees of freedom (DF), T‐value, and corre [file ECE3-16-e73508-s001.docx]

We fitted separate mixed effects models to estimate the average δ¹⁵N and δ¹³C response, respectively averaged across all tadpole taxa (Equation 1). As such, tadpole taxon identity (i.e. genus) was included as a random effect. Fixed effects included sampling period (BW vs AW) and pond identity (A vs H), and their interaction. Models were fitted using the R package *lme4* (Bates et al. 2015). Results are included in the main manuscript (see Figure 2), and observed values for each tadpole taxon and pond site are shown below in Figure SI1.

1. $Y$*_ij_* = β_0_ + $\beta$_1_ × period*_ij_* + $\beta$_2_ × pond*_ij_* + $\beta$_3_ × period*_ij_* pond*_ij_* + $u$_j_ + ε_i_

Where:

- $Y$*_i_* is the isotopic response (δ¹⁵N or δ¹³C) for observation *i* and genus *j*;
- $\beta$_0_ is the intercept ;
- $\beta$_1_, $\beta$_2_, and $\beta$_3_ are the coefficients for the fixed effects of sampling period, pond identity, and their interaction, respectively;
- $u$_j_ is the random intercept for tadpole genus *j*, with u_j_ ∼ $N$(0,$\sigma_{u}^{2}$);
- ε_ij_ is the residual error term, with ε_ij_ ∼ $N$(0,σ^2^).

Initial model diagnostics indicated unequal variance in residuals across all levels of each categorical predictor, so we incorporated observation-level weights inversely proportional to the estimated residual variance for each combination of predictors (period and site). Weights ($\omega_{i}$) were calculated as: $\omega_{i}=\frac{1}{\hat{\sigma}_{g}^{2}}$, where $\hat{\sigma}_{g}^{2}$ is the estimated residual variance for group $g$, defined by unique combinations of period and site. Residual variance estimates were obtained from an unweighted model and assigned to each observation based on its respective group.

Following the same approach, we estimated the δ¹⁵N and δ¹³C responses for each tadpole taxon by fitting separate linear regression models. We added tadpole body length as an additional predictor, despite incomplete developmental stage information for all observations, in an attempt to test for potential ontogenetic effects (Equation 2). Note that for the tadpoles *Hemisus*, *Hyperolius*, and *Phrynobatrachus*, for which we did not have a large enough sample size for Pond A in both sampling periods (N < 3), we only included observations for Pond H and excluded pond identity as a predictor. Results are summarized in Table S1 and S2.

1. $Y$*_i_* = β_0_ + $\beta$_1_ × body size*_i_* + $\beta$_2_ × period*_i_* + $\beta$_3_ × pond*_i_* + $\beta$_4_ × period*_i_* pond*_i_* + ε_i_

Where:

- $Y$*_i_* is the isotopic response (δ¹⁵N or δ¹³C) for observation *i*;
- $\beta$_0_ is the intercept;
- $\beta$_1_, $\beta$_2_, $\beta$_3_, and $\beta$_4_ are the coefficients for the predictors body length, sampling period, pond identity, and the interaction of period and pond identity, respectively;
- ε_i_ is the residual error term, with ε_i_ ∼ $N$(0,σ^2^).

For each tadpole taxon and each pond site, we estimated the BW isotopic baseline and AW change using the R package *emmeans* (Lenth, 2025). Estimates were obtained for each taxon with body size as the mean observed value in our dataset. All estimates and test results are summarized in Table S3.

**Figure S1.** Observed isotope values of δ¹³C (x-axis) and δ¹⁵N (y-axis) for each taxon and pond site. Pre-war (BW) and post-war (AW) samples are shown in yellow and green, respectively.


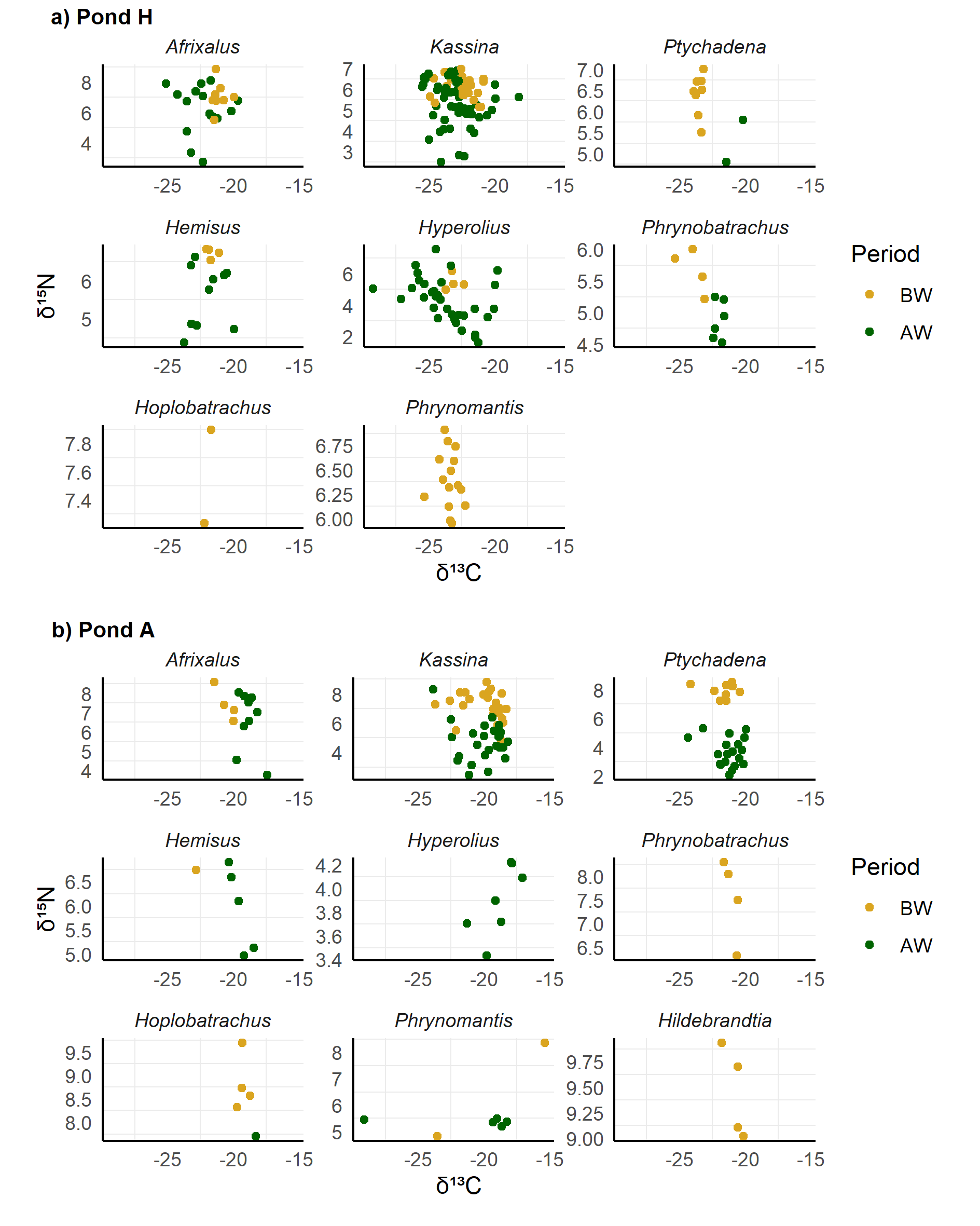


**Table S1.** Summary of linear models to estimate the effects of body size (cm), sampling period (BW vs AW), pond site (A vs H), and the interaction of sampling period and pond site, on the δ¹⁵N signature of each tadpole taxon. Significant predictors are highlighted in bold (α = 0.05). For each model parameter we provide the estimate and standard error (SE). We tested the significance of each model predictor using a Wald test, and we performed a log-likelihood ratio (LLR) test to compare each full model to the null model with intercept only, with corresponding degrees of freedom (DF), R², and change in AIC.

|  | **Predictor** | **Estimate** | **SE** | **T-value** | **LLR** | **DF** | **P-value** | **R²** | **AIC** | **ΔAIC** |
| --- | --- | --- | --- | --- | --- | --- | --- | --- | --- | --- |
| *Afrixalus* spp. |  |  |  |  | -16.75 | 32, 36 | 0.003 | 0.66 | 123.7 | -7.2 |
|  | Intercept† | 9.43 | 0.82 | 11.43 |  | 32 | <0.001 |  |  |  |
|  | **bodylength** | -3.66 | 1.13 | -3.23 |  | 32 | 0.003 |  |  |  |
|  | periodAW | -0.11 | 0.56 | -0.19 |  | 32 | 0.848 |  |  |  |
|  | pondA | 0.13 | 0.59 | 0.22 |  | 32 | 0.824 |  |  |  |
|  | periodAW:pondA | 0.03 | 0.77 | 0.04 |  | 32 | 0.969 |  |  |  |
| *Kassina* spp. |  |  |  |  | -80.82 | 121, 125 | <0.001 | 0.60 | 347.3 | -56.3 |
|  | Intercept† | 6.44 | 0.23 | 28.10 |  | 121 | <0.001 |  |  |  |
|  | bodylength | -0.39 | 0.24 | -1.67 |  | 121 | 0.098 |  |  |  |
|  | **perioddN** | -0.64 | 0.19 | -3.28 |  | 121 | 0.001 |  |  |  |
|  | **pondA** | 1.05 | 0.23 | 4.65 |  | 121 | <0.001 |  |  |  |
|  | **periodAW:pondA** | -1.71 | 0.38 | -4.54 |  | 121 | <0.001 |  |  |  |
| *Ptychadena* spp. |  |  |  |  | -261.77 | 34, 38 | <0.001 | 0.12 | 89.9 | -75.4 |
|  | Intercept† | 5.81 | 0.68 | 8.49 |  | 34 | <0.001 |  |  |  |
|  | bodylength | 0.82 | 0.91 | 0.90 |  | 34 | 0.375 |  |  |  |
|  | periodAW | -1.33 | 0.76 | -1.75 |  | 34 | 0.090 |  |  |  |
|  | **pondA** | 1.38 | 0.31 | 4.48 |  | 34 | <0.001 |  |  |  |
|  | **periodAW:pondA** | -2.91 | 0.83 | -3.50 |  | 34 | 0.001 |  |  |  |
| *Hemisus maromoratus* |  |  |  |  | -18.17 | 11, 13 | <0.001 | 0.39 | 25.0 | -9.3 |
|  | Intercept† | 6.33 | 1.07 | 5.93 |  | 11 | <0.001 |  |  |  |
|  | bodylength | 0.33 | 0.91 | 0.37 |  | 11 | 0.721 |  |  |  |
|  | **periodAW** | -1.13 | 0.27 | -4.17 |  | 11 | 0.002 |  |  |  |
| *Hyperolius* spp. |  |  |  |  | -13.92 | 34, 36 | 0.001 | 0.72 | 126.8 | -8.4 |
|  | Intercept† | 4.59 | 0.65 | 7.05 |  | 34 | <0.001 |  |  |  |
|  | bodylength | 1.02 | 0.72 | 1.41 |  | 34 | 0.167 |  |  |  |
|  | **periodAW** | -1.22 | 0.34 | -3.57 |  | 34 | 0.001 |  |  |  |
| *Phrynobatrachus* spp. |  |  |  |  | -15.10 | 7, 9 | 0.001 | 0.35 | 11.0 | -6.6 |
|  | Intercept† | 5.31 | 0.62 | 8.55 |  | 7 | <0.001 |  |  |  |
|  | bodylength | 0.80 | 1.38 | 0.58 |  | 7 | 0.579 |  |  |  |
|  | **periodAW** | -0.97 | 0.39 | -2.48 |  | 7 | 0.042 |  |  |  |

† Intercept taken for body length set to 0 cm, pond H and sampling period BW.

**Table S2.** Summary of linear models to estimate the effects of body size (cm), sampling period (BW vs AW), pond site (A vs H), and the interaction of sampling period and pond site, on the δ¹³C signature of each tadpole taxon. Significant predictors are highlighted in bold (α = 0.05). For each model parameter we provide the estimate and standard error (SE). We tested the significance of each model predictor using a Wald test, and we performed a log-likelihood ratio (LLR) test to compare each full model to the null model with intercept only, with corresponding degrees of freedom (DF), R², and change in AIC.

|  | **Predictor** | **Estimate** | **SE** | **T-value** | **LLR** | **DF** | **P-value** | **R²** | **AIC** | **ΔAIC** |
| --- | --- | --- | --- | --- | --- | --- | --- | --- | --- | --- |
| *Afrixalus* spp. |  |  |  |  | -71.54 | 32, 36 | <0.001 | 0.35 | 117.8 | -31.3 |
|  | Intercept† | -21.28 | 0.77 | -27.71 |  | 32 | <0.001 |  |  |  |
|  | bodylength | 0.31 | 1.03 | 0.30 |  | 32 | 0.766 |  |  |  |
|  | **periodAW** | -1.33 | 0.52 | -2.57 |  | 32 | 0.015 |  |  |  |
|  | pondA | 0.57 | 0.67 | 0.86 |  | 32 | 0.397 |  |  |  |
|  | **periodAW:pondA** | 2.94 | 0.80 | 3.65 |  | 32 | <0.001 |  |  |  |
| *Kassina* spp. |  |  |  |  | -241.77 | 121, 125 | <0.001 | 0.51 | 456.4 | -77.0 |
|  | Intercept† | -23.15 | 0.50 | -46.70 |  | 121 | <0.001 |  |  |  |
|  | bodylength | 0.78 | 0.47 | 1.66 |  | 121 | 0.100 |  |  |  |
|  | **periodAW** | -0.75 | 0.38 | -1.98 |  | 121 | 0.050 |  |  |  |
|  | **pondA** | 2.54 | 0.41 | 6.22 |  | 121 | <0.001 |  |  |  |
|  | periodAW:pondA | 0.48 | 0.54 | 0.88 |  | 121 | 0.378 |  |  |  |
| *Ptychadena* spp. |  |  |  |  | -33.08 | 34, 38 | <0.001 | 0.48 | 112.9 | -20.7 |
|  | Intercept† | -25.07 | 1.03 | -24.35 |  | 34 | <0.001 |  |  |  |
|  | bodylength | 2.13 | 1.33 | 1.60 |  | 34 | 0.119 |  |  |  |
|  | periodAW | 2.12 | 0.83 | 2.56 |  | 34 | 0.015 |  |  |  |
|  | **pondA** | 1.40 | 0.54 | 2.58 |  | 34 | 0.014 |  |  |  |
|  | periodAW:pondA | -1.62 | 0.95 | -1.70 |  | 34 | 0.098 |  |  |  |
| *Hemisus maromoratus* |  |  |  |  | -0.47 | 11, 13 | 0.851 | 0.97 | 49.7 | 3.6 |
|  | Intercept† | -21.11 | 2.68 | -7.87 |  | 11 | <0.001 |  |  |  |
|  | bodylength | -0.48 | 2.24 | -0.21 |  | 11 | 0.834 |  |  |  |
|  | periodAW | -0.37 | 0.72 | -0.52 |  | 11 | 0.616 |  |  |  |
| *Hyperolius* spp. |  |  |  |  | -15.30 | 34, 36 | 0.155 | 0.90 | 162.1 | 0.1 |
|  | Intercept† | -21.32 | 1.40 | -15.23 |  | 34 | <0.001 |  |  |  |
|  | bodylength | -2.13 | 1.14 | -1.87 |  | 34 | 0.071 |  |  |  |
|  | periodAW | -0.39 | 1.08 | -0.36 |  | 34 | 0.721 |  |  |  |
| *Phrynobatrachus* spp. |  |  |  |  | -9.73 | 7, 9 | <0.001 | 0.23 | 23.9 | -10.8 |
|  | Intercept† | -22.09 | 1.20 | -18.46 |  | 7 | <0.001 |  |  |  |
|  | bodylength | -4.22 | 2.67 | -1.58 |  | 7 | 0.158 |  |  |  |
|  | periodAW | 2.90 | 0.75 | 3.86 |  | 7 | 0.006 |  |  |  |

† Intercept taken for body length set to 0 cm, pond H and sampling period BW.

**Table S3.** Significance of each AW change in isotopic levels (δ¹⁵N and δ¹³C) for each pond (A vs H) and tadpole taxon, compared to the BW baseline. Significant AW shifts are highlighted in bold (α = 0.05). For each test we provide the standard error (SE), number of degrees of freedom (DF), T-value, and corresponding P-value. For taxa with low sample size, i.e. *H. ornata*, *H. occipitalis*, and *P. microps*, BW and AW estimates correspond to the mean values.

|  | **Isotope** | **Pond** | **BW baseline** | **AW change** | **SE** | **DF** | **T-value** | **P-value** |
| --- | --- | --- | --- | --- | --- | --- | --- | --- |
| *Afrixalus* spp. | δ¹⁵N | H | 6.63 | -0.11 | 0.563 | 32 | -0.194 | 0.848 |
|  |  | A | 6.76 | -0.08 | 0.625 | 32 | -0.126 | 0.901 |
|  | δ¹³C | H | -21.04 | **-1.33** | 0.518 | 32 | -2.567 | 0.015 |
|  |  | A | -20.47 | **1.61** | 0.685 | 32 | 2.352 | 0.025 |
| *Kassina* spp. | δ¹⁵N | H | 6.03 | **-0.64** | 0.194 | 121 | -3.279 | 0.001 |
|  |  | A | 7.08 | **-2.34** | 0.333 | 121 | -7.035 | <0.001 |
|  | δ¹³C | H | -22.35 | **-0.75** | 0.380 | 121 | -1.984 | 0.050 |
|  |  | A | -19.81 | -0.28 | 0.422 | 121 | -0.652 | 0.515 |
| *Ptychadena* spp. | δ¹⁵N | H | 6.53 | -1.33 | 0.762 | 34 | -1.746 | 0.090 |
|  |  | A | 7.90 | **-4.24** | 0.280 | 34 | -15.154 | <0.001 |
|  | δ¹³C | H | -23.20 | **+2.12** | 0.830 | 34 | 2.556 | 0.015 |
|  |  | A | -21.80 | +0.50 | 0.382 | 34 | 1.320 | 0.196 |
| *Hemisus marmoratus* | δ¹⁵N | H | 6.72 | **-1.13** | 0.271 | 11 | -4.171 | 0.002 |
|  | δ¹³C | H | -21.66 | -0.37 | 0.717 | 11 | -0.516 | 0.616 |
| *Hyperolius* spp. | δ¹⁵N | H | 5.52 | **-1.22** | 0.340 | 34 | -3.570 | 0.001 |
|  | δ¹³C | H | -23.27 | -0.39 | 1.075 | 34 | -0.360 | 0.721 |
| *Phrynobatrachus* spp. | δ¹⁵N | H | 5.73 | **-0.97** | 0.392 | 7 | -2.484 | 0.042 |
|  | δ¹³C | H | -24.28 | **+2.90** | 0.751 | 7 | 3.856 | 0.006 |
| *Hildebrandtia ornata* | δ¹⁵N | A | 9.44 | - |  |  |  |  |
|  | δ¹³C | A | -20.77 | - |  |  |  |  |
| *Hoplobatrachus occipitalis* | δ¹⁵N | H | 7.57 | - |  |  |  |  |
|  |  | A | 8.83 | -1.13 |  |  |  |  |
|  | δ¹³C | H | -21.95 | - |  |  |  |  |
|  |  | A | -19.28 | +0.98 |  |  |  |  |
| *Phrynomantis microps* | δ¹⁵N | H | 6.39 | - |  |  |  |  |
|  |  | A | 6.59 | -1.22 |  |  |  |  |
|  | δ¹³C | H | -23.43 | - |  |  |  |  |
|  |  | A | -19.44 | -1.43 |  |  |  |  |

Finally, we repeated the same approach to investigate isotopic differences between sampling years, thus disaggregating data in each sampling period. However, we fitted separate models for each pond site (Equation 3). Results are summarized in Table SI4 and SI5, and observed values for each tadpole taxon and year are shown below in Figure SI2 and Figure SI3.

1. $Y$*_i_* = β_0_ + $\beta$_1_ × body size*_i_* + $\beta$_2_ × year*_i_* + ε_i_

Where:

- $Y$*_i_* is the isotopic response (δ¹⁵N or δ¹³C) for observation *i*;
- $\beta$_0_ is the intercept ;
- $\beta$_1_ and $\beta$_2_ are the coefficients for the predictors body length and sampling year, respectively;
- ε_i_ is the residual error term, with ε_i_ ∼ $N$(0,σ^2^).

**Figure S2.** Observed isotope values of δ¹⁵N and corresponding violin plots for each taxon, sampling year, and pond site.


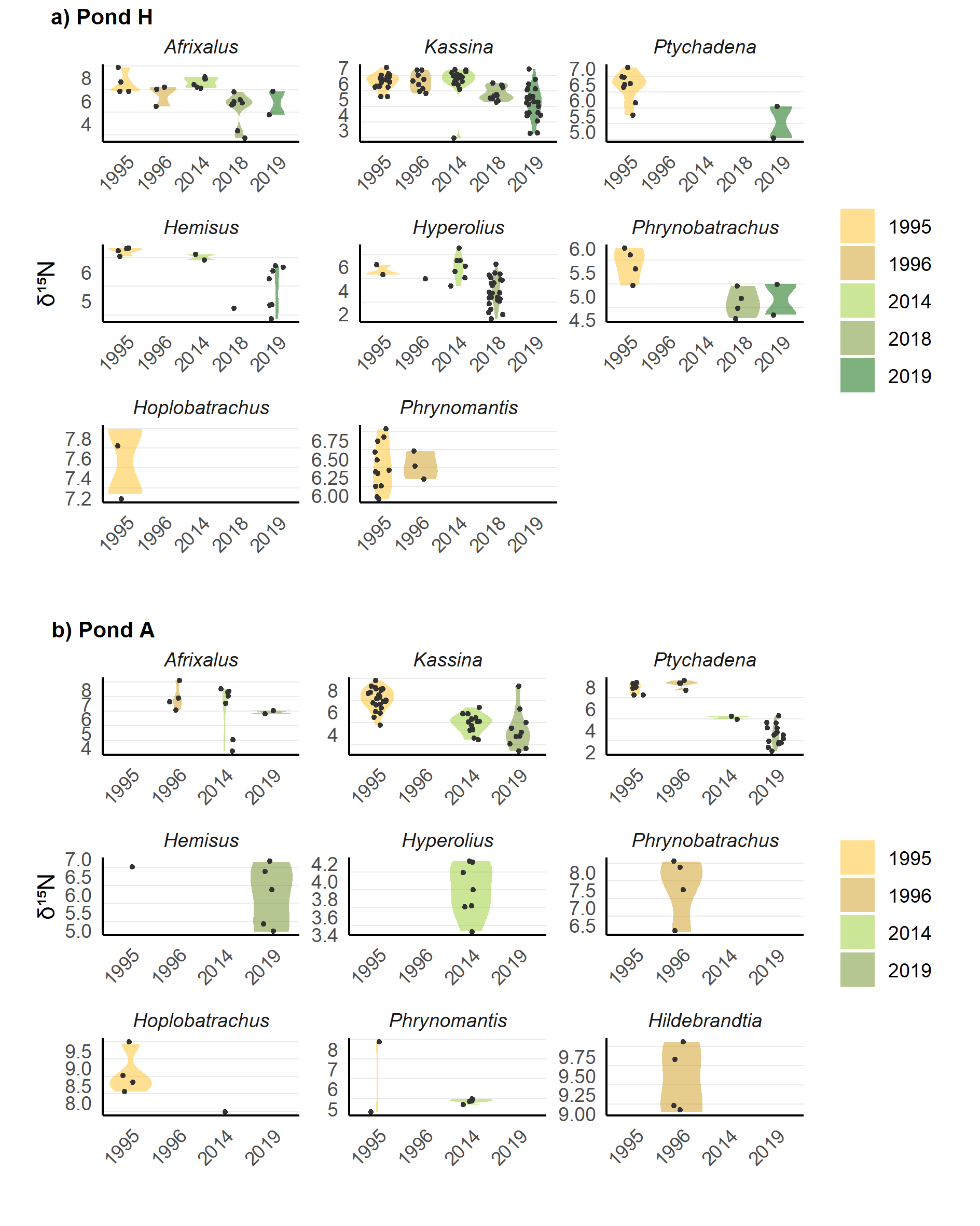


**Figure S3.** Observed isotope values of δ^13^C and corresponding violin plots for each taxon, sampling year, and pond site.


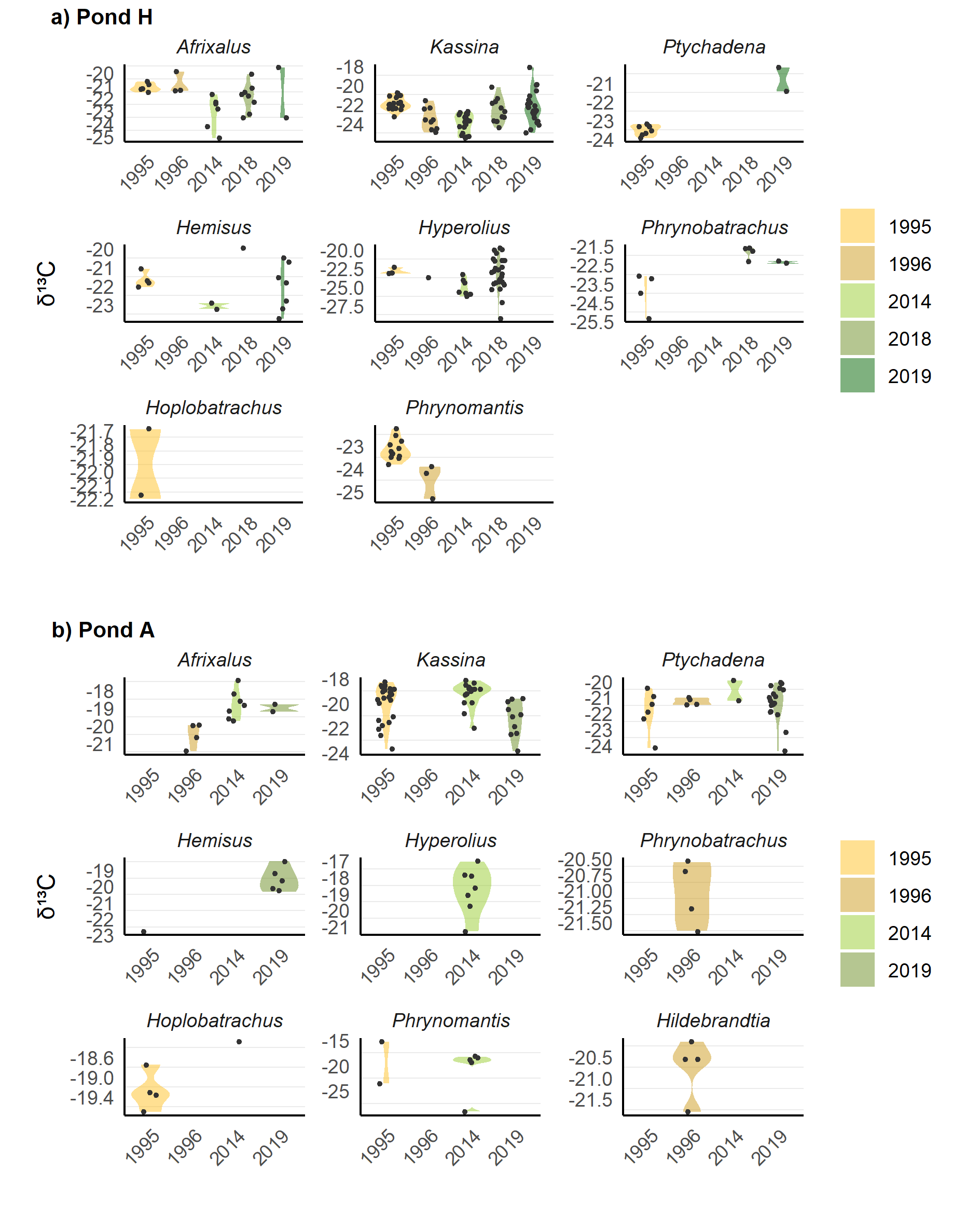


**Table S4.** Summary of linear models to estimate the effects of body size (cm) and sampling year on the δ¹⁵N signature of each tadpole taxon at each pond site. Significant predictors are highlighted in bold (α = 0.05). For each model parameter we provide the estimate and standard error (SE). We tested the significance of each model predictor using a Wald test, and we performed a log-likelihood ratio (LLR) test to compare each full model to the null model with intercept only, with corresponding degrees of freedom (DF), R², and change in AIC.

|  | **Pond** | **Predictor** | **Estimate** | **SE** | **T-value** | **LLR** | **DF** | **P-value** | **R²** | **AIC** | **ΔAIC** |
| --- | --- | --- | --- | --- | --- | --- | --- | --- | --- | --- | --- |
| *Afrixalus* spp. | **H** |  |  |  |  | -22.30 | 17, 21 | <0.001 | 0.42 | 62.6 | -11.2 |
|  |  | Intercept† | 6.65 | 0.78 | 8.57 |  | 17 | <0.001 |  |  |  |
|  |  | bodylength | 1.08 | 1.01 | 1.08 |  | 17 | 0.296 |  |  |  |
|  |  | year1996 | -0.76 | 0.66 | -1.15 |  | 17 | 0.266 |  |  |  |
|  |  | year2014 | 0.20 | 0.44 | 0.45 |  | 17 | 0.660 |  |  |  |
|  |  | **year2018** | -2.45 | 0.69 | -3.56 |  | 17 | 0.002 |  |  |  |
|  | **A** |  |  |  |  | -19.13 | 8, 10 | <0.001 | 0.31 | 35.7 | -8.7 |
|  |  | Intercept† | 11.38 | 1.15 | 9.87 |  | 8 | <0.001 |  |  |  |
|  |  | **bodylength** | -6.76 | 1.68 | -4.03 |  | 8 | 0.004 |  |  |  |
|  |  | year2014 | 0.96 | 0.81 | 1.18 |  | 8 | 0.271 |  |  |  |
| *Hyperolius* spp. | **H** |  |  |  |  | -33.22 | 32, 35 | <0.001 | 0.50 | 113.7 | -19.1 |
|  |  | Intercept† | 5.28 | 0.70 | 7.59 |  | 32 | <0.001 |  |  |  |
|  |  | bodylength | 0.36 | 0.68 | 0.52 |  | 32 | 0.605 |  |  |  |
|  |  | year2014 | 0.30 | 0.49 | 0.61 |  | 32 | 0.548 |  |  |  |
|  |  | **year2018** | -1.73 | 0.37 | -4.61 |  | 32 | <0.001 |  |  |  |
| *Kassina* spp. | **H** |  |  |  |  | -83.60 | 71, 76 | <0.001 | 0.46 | 161.4 | -49.5 |
|  |  | Intercept† | 6.93 | 0.20 | 35.10 |  | 71 | <0.001 |  |  |  |
|  |  | **bodylength** | -1.09 | 0.23 | -4.70 |  | 71 | <0.001 |  |  |  |
|  |  | year1996 | 0.31 | 0.31 | 0.99 |  | 71 | 0.327 |  |  |  |
|  |  | year2014 | 0.42 | 0.26 | 1.59 |  | 71 | 0.116 |  |  |  |
|  |  | year2018 | -0.22 | 0.18 | -1.23 |  | 71 | 0.221 |  |  |  |
|  |  | **year2019** | -0.87 | 0.23 | -3.74 |  | 71 | <0.001 |  |  |  |
|  | **A** |  |  |  |  | -66.29 | 45, 48 | <0.001 | 0.41 | 150.1 | -37.7 |
|  |  | Intercept† | 7.10 | 0.45 | 15.67 |  | 45 | <0.001 |  |  |  |
|  |  | bodylength | 0.07 | 0.48 | 0.14 |  | 45 | 0.889 |  |  |  |
|  |  | **year2014** | -2.21 | 0.31 | -7.16 |  | 45 | <0.001 |  |  |  |
|  |  | **year2019** | -2.77 | 0.61 | -4.55 |  | 45 | <0.001 |  |  |  |
| *Phrynobatrachus* spp. | **H** |  |  |  |  | -25.58 | 5, 7 | <0.001 | 0.19 | 6.0 | -9.3 |
|  |  | Intercept† | 2.39 | 1.56 | 1.54 |  | 5 | 0.185 |  |  |  |
|  |  | bodylength | 7.57 | 3.60 | 2.11 |  | 5 | 0.089 |  |  |  |
|  |  | **year2018** | -2.08 | 0.64 | -3.27 |  | 5 | 0.022 |  |  |  |
| *Phrynomantis microps* | **H** |  |  |  |  | -5.84 | 12, 14 | 0.068 | 0.69 | 6.7 | -1.6 |
|  |  | Intercept† | 5.70 | 0.31 | 18.47 |  | 12 | <0.001 |  |  |  |
|  |  | **bodylength** | 0.95 | 0.41 | 2.32 |  | 12 | 0.039 |  |  |  |
|  |  | year1996 | -0.14 | 0.17 | -0.83 |  | 12 | 0.423 |  |  |  |
| *Ptychadena* spp. | **A** |  |  |  |  | -288.22 | 23, 26 | <0.001 | 0.08 | 64.3 | -63.9 |
|  |  | Intercept† | 7.64 | 1.14 | 6.68 |  | 23 | <0.001 |  |  |  |
|  |  | bodylength | 0.20 | 1.28 | 0.15 |  | 23 | 0.880 |  |  |  |
|  |  | year1996 | 0.36 | 0.41 | 0.86 |  | 23 | 0.398 |  |  |  |
|  |  | **year2019** | -4.32 | 0.30 | -14.45 |  | 23 | <0.001 |  |  |  |

† Intercept taken for body length set to 0 cm and year 1995 (1996 for *Afrixalus* at pond A).

**Table S5.** Summary of linear models to estimate the effects of body size (cm) and sampling year on the δ^13^C signature of each tadpole taxon at each pond site. Significant predictors are highlighted in bold (α = 0.05). For each model parameter we provide the estimate and standard error (SE). We tested the significance of each model predictor using a Wald test, and we performed a log-likelihood ratio (LLR) test to compare each full model to the null model with intercept only, with corresponding degrees of freedom (DF), R², and change in AIC.

|  | **Pond** | **Predictor** | **Estimate** | **SE** | **T-value** | **LLR** | **DF** | **P-value** | **R²** | **AIC** | **ΔAIC** |
| --- | --- | --- | --- | --- | --- | --- | --- | --- | --- | --- | --- |
| *Afrixalus* spp. | **H** |  |  |  |  | -21.91 | 17, 21 | <0.001 | 0.33 | 59.1 | -16.1 |
|  |  | Intercept† | -18.23 | 0.93 | -19.54 |  | 17 | <0.001 |  |  |  |
|  |  | **bodylength** | -4.43 | 1.30 | -3.42 |  | 17 | 0.003 |  |  |  |
|  |  | year1996 | 0.02 | 0.59 | 0.04 |  | 17 | 0.967 |  |  |  |
|  |  | **year2014** | -1.87 | 0.49 | -3.83 |  | 17 | 0.001 |  |  |  |
|  |  | year2018 | 0.54 | 0.60 | 0.90 |  | 17 | 0.380 |  |  |  |
|  | **A** |  |  |  |  | -8.32 | 8, 10 | <0.001 | 0.36 | 29.7 | -7.3 |
|  |  | Intercept† | -21.42 | 0.85 | -25.14 |  | 8 | <0.001 |  |  |  |
|  |  | bodylength | 1.53 | 1.30 | 1.17 |  | 8 | 0.275 |  |  |  |
|  |  | year2014 | 1.33 | 0.58 | 2.28 |  | 8 | 0.052 |  |  |  |
| *Hyperolius* spp. | **H** |  |  |  |  | -30.72 | 32, 35 | 0.048 | 0.80 | 156.7 | -2.0 |
|  |  | Intercept† | -21.19 | 1.56 | -13.62 |  | 32 | <0.001 |  |  |  |
|  |  | bodylength | -1.85 | 1.14 | -1.62 |  | 32 | 0.116 |  |  |  |
|  |  | year2014 | -2.03 | 1.36 | -1.49 |  | 32 | 0.147 |  |  |  |
|  |  | year2018 | -0.43 | 1.20 | -0.36 |  | 32 | 0.725 |  |  |  |
| *Kassina* spp. | **H** |  |  |  |  | -53.42 | 71, 76 | <0.001 | 0.65 | 253.6 | -22.6 |
|  |  | Intercept† | -22.68 | 0.51 | -44.68 |  | 71 | <0.001 |  |  |  |
|  |  | bodylength | 0.98 | 0.54 | 1.81 |  | 71 | 0.075 |  |  |  |
|  |  | **year1996** | -1.86 | 0.51 | -3.61 |  | 71 | <0.001 |  |  |  |
|  |  | **year2014** | -2.47 | 0.45 | -5.44 |  | 71 | <0.001 |  |  |  |
|  |  | **year2018** | -1.18 | 0.53 | -2.23 |  | 71 | 0.029 |  |  |  |
|  |  | year2019 | -0.85 | 0.45 | -1.89 |  | 71 | 0.063 |  |  |  |
|  | **A** |  |  |  |  | -25.60 | 45, 48 | 0.003 | 0.76 | 173.6 | -7.5 |
|  |  | Intercept† | -20.67 | 0.63 | -32.87 |  | 45 | <0.001 |  |  |  |
|  |  | bodylength | 0.85 | 0.67 | 1.25 |  | 45 | 0.216 |  |  |  |
|  |  | year2014 | 0.48 | 0.46 | 1.05 |  | 45 | 0.299 |  |  |  |
|  |  | **year2019** | -1.45 | 0.52 | -2.78 |  | 45 | 0.008 |  |  |  |
| *Phrynobatrachus* spp. | **H** |  |  |  |  | -11.30 | 5, 7 | <0.001 | 0.07 | 12.8 | -17.2 |
|  |  | **Intercept†** | -12.77 | 2.83 | -4.51 |  | 5 | 0.006 |  |  |  |
|  |  | **bodylength** | -25.81 | 6.55 | -3.94 |  | 5 | 0.011 |  |  |  |
|  |  | **year2018** | 6.42 | 1.14 | 5.64 |  | 5 | 0.002 |  |  |  |
| *Phrynomantis microps* | **H** |  |  |  |  | -5.83 | 12, 14 | <0.001 | 0.23 | 18.3 | -18.0 |
|  |  | Intercept† | -21.71 | 0.45 | -48.74 |  | 12 | <0.001 |  |  |  |
|  |  | **bodylength** | -1.99 | 0.59 | -3.37 |  | 12 | 0.006 |  |  |  |
|  |  | **year1996** | -0.97 | 0.27 | -3.66 |  | 12 | 0.003 |  |  |  |
| *Ptychadena* spp. | **A** |  |  |  |  | -3.65 | 23, 26 | 0.363 | 0.88 | 85.9 | 2.5 |
|  |  | Intercept† | -24.40 | 1.82 | -13.39 |  | 23 | <0.001 |  |  |  |
|  |  | bodylength | 2.87 | 2.01 | 1.43 |  | 23 | 0.167 |  |  |  |
|  |  | year1996 | 0.05 | 0.79 | 0.06 |  | 23 | 0.951 |  |  |  |
|  |  | year2019 | 0.50 | 0.51 | 0.99 |  | 23 | 0.335 |  |  |  |

† Intercept taken for body length set to 0 cm and year 1995 (1996 for *Afrixalus* at pond A).

We conducted post-hoc tests to compare pairwise differences between years using the R package *emmeans* (Lenth, 2025). Results are summarized in Table SI6 and SI7.

**Table S6.** Post-hoc test to compare pairwise year differences in δ¹⁵N for each tadpole taxon at each pond site (A and H). Significant pairwise differences are highlighted in bold (α = 0.05). For each test we provide the standard error (SE), number of degrees of freedom (DF), T-ratio, and corresponding P-value

|  | **Pond** | **Years** | **Estimate** | **SE** | **DF** | **T-ratio** | **P-value** |
| --- | --- | --- | --- | --- | --- | --- | --- |
| *Afrixalus* spp. | **H** | 1995 - 1996 | -0.759 | 0.659 | 17 | -1.151 | 0.664 |
|  |  | 1995 - 2014 | 0.196 | 0.438 | 17 | 0.448 | 0.969 |
|  |  | **1995 - 2018** | **-2.449** | **0.689** | **17** | **-3.556** | **0.012** |
|  |  | 1996 - 2014 | 0.955 | 0.563 | 17 | 1.696 | 0.356 |
|  |  | 1996 - 2018 | -1.690 | 0.792 | 17 | -2.134 | 0.182 |
|  |  | **2014 - 2018** | **-2.645** | **0.586** | **17** | **-4.510** | **0.002** |
|  | **A** | 1996 - 2014 | 0.962 | 0.813 | 8 | 1.183 | 0.271 |
| *Hyperolius* spp. | **H** | 1995 - 2014 | 0.300 | 0.495 | 32 | 0.607 | 0.817 |
|  |  | **1995 - 2018** | **-1.727** | **0.375** | **32** | **-4.605** | **<0.001** |
|  |  | **2014 - 2018** | **-2.027** | **0.469** | **32** | **-4.325** | **<0.001** |
| *Kassina* spp. | **H** | 1995 - 1996 | 0.306 | 0.311 | 71 | 0.986 | 0.861 |
|  |  | 1995 - 2014 | 0.415 | 0.261 | 71 | 1.590 | 0.509 |
|  |  | 1995 - 2018 | -0.224 | 0.181 | 71 | -1.235 | 0.731 |
|  |  | **1995 - 2019** | **-0.869** | **0.233** | **71** | **-3.736** | **0.003** |
|  |  | 1996 - 2014 | 0.109 | 0.374 | 71 | 0.291 | 0.998 |
|  |  | 1996 - 2018 | -0.530 | 0.318 | 71 | -1.666 | 0.462 |
|  |  | **1996 - 2019** | **-1.175** | **0.352** | **71** | **-3.342** | **0.011** |
|  |  | 2014 - 2018 | -0.639 | 0.262 | 71 | -2.442 | 0.116 |
|  |  | **2014 - 2019** | **-1.284** | **0.302** | **71** | **-4.253** | **<0.001** |
|  |  | **2018 - 2019** | **-0.645** | **0.224** | **71** | **-2.881** | **0.041** |
|  | **A** | **1995 - 2014** | **-2.210** | **0.309** | **45** | **-7.156** | **<0.001** |
|  |  | **1995 - 2019** | **-2.773** | **0.609** | **45** | **-4.553** | **<0.001** |
|  |  | 2014 - 2019 | -0.564 | 0.607 | 45 | -0.929 | 0.625 |
| *Phrynobatrachus* spp. | **H** | **1995 - 2018** | -2.080 | 0.636 | 5 | -3.271 | 0.022 |
| *Phrynomantis microps* | **H** | 1995 - 1996 | -0.144 | 0.174 | 12 | -0.829 | 0.423 |
| *Ptychadena* spp. | **A** | 1995 - 1996 | 0.357 | 0.415 | 23 | 0.862 | 0.669 |
|  |  | **1995 - 2019** | **-4.317** | **0.299** | **23** | **-14.453** | **<0.001** |
|  |  | **1996 - 2019** | **-4.675** | **0.412** | **23** | **-11.339** | **<0.001** |

**Table S7.** Post-hoc test to compare pairwise year differences in δ^13^C for each tadpole taxon at each pond site (A and H). Significant pairwise differences are highlighted in bold (α = 0.05). For each test we provide the standard error (SE), number of degrees of freedom (DF), T-ratio, and corresponding P-value

|  | **Pond** | **Years** | **Estimate** | **SE** | **DF** | **T-ratio** | **P-value** |
| --- | --- | --- | --- | --- | --- | --- | --- |
| *Afrixalus* spp. | **H** | 1995 - 1996 | 0.025 | 0.590 | 17 | 0.042 | 1.000 |
|  |  | **1995 - 2014** | **-1.866** | **0.487** | **17** | **-3.830** | **0.007** |
|  |  | 1995 - 2018 | 0.539 | 0.599 | 17 | 0.900 | 0.805 |
|  |  | **1996 - 2014** | **-1.891** | **0.575** | **17** | **-3.288** | **0.020** |
|  |  | 1996 - 2018 | 0.514 | 0.706 | 17 | 0.728 | 0.884 |
|  |  | **2014 - 2018** | **2.405** | **0.565** | **17** | **4.258** | **0.003** |
|  | **A** | 1996 - 2014 | 1.328 | 0.582 | 8 | 2.283 | 0.052 |
| *Hyperolius* spp. | **H** | 1995 - 2014 | -2.028 | 1.363 | 32 | -1.488 | 0.310 |
|  |  | 1995 - 2018 | -0.427 | 1.201 | 32 | -0.355 | 0.933 |
|  |  | 2014 - 2018 | 1.601 | 0.853 | 32 | 1.876 | 0.162 |
| *Kassina* spp. | **H** | 1995 - 1996 | -1.856 | 0.513 | 71 | -3.615 | 0.005 |
|  |  | **1995 - 2014** | **-2.466** | **0.454** | **71** | **-5.437** | **<0.001** |
|  |  | 1995 - 2018 | -1.183 | 0.530 | 71 | -2.234 | 0.179 |
|  |  | 1995 - 2019 | -0.852 | 0.451 | 71 | -1.891 | 0.331 |
|  |  | 1996 - 2014 | -0.610 | 0.495 | 71 | -1.234 | 0.732 |
|  |  | 1996 - 2018 | 0.673 | 0.548 | 71 | 1.226 | 0.736 |
|  |  | 1996 - 2019 | 1.004 | 0.479 | 71 | 2.097 | 0.233 |
|  |  | 2014 - 2018 | 1.283 | 0.466 | 71 | 2.750 | 0.057 |
|  |  | **2014 - 2019** | **1.614** | **0.384** | **71** | **4.198** | **<0.001** |
|  |  | 2018 - 2019 | 0.331 | 0.438 | 71 | 0.756 | 0.942 |
|  | **A** | 1995 - 2014 | 0.485 | 0.461 | 45 | 1.051 | 0.549 |
|  |  | **1995 - 2019** | **-1.447** | **0.520** | **45** | **-2.781** | **0.021** |
|  |  | **2014 - 2019** | **-1.932** | **0.547** | **45** | **-3.531** | **0.003** |
| *Phrynobatrachus* spp. | **H** | **1995 - 2018** | **6.421** | **1.139** | **5** | **5.636** | **0.002** |
| *Phrynomantis microps* | **H** | **1995 - 1996** | **-0.974** | **0.266** | **12** | **-3.656** | **0.003** |
| *Ptychadena* spp. | **A** | 1995 - 1996 | 0.049 | 0.792 | 23 | 0.062 | 0.998 |
|  |  | 1995 - 2019 | 0.500 | 0.508 | 23 | 0.986 | 0.593 |
|  |  | 1996 - 2019 | 0.451 | 0.707 | 23 | 0.638 | 0.801 |

**REFERENCES**

Bates, D., Mächler, M., Bolker, B., & Walker, S. (2015). Fitting linear mixed-effects models using *lme4*. *Journal of Statistical Software*, *67*(1), 1–48.

Lenth, R. (2025). emmeans: Estimated Marginal Means, aka Least-Squares Means. R package version 1.11.2-80001, <https://www.rvlenth.github.io/emmeans/>.
